# Weight Science: Evaluating the Evidence for a Paradigm Shift

**DOI:** 10.1186/1475-2891-10-9

**Published:** 2011-01-24

**Authors:** Linda Bacon, Lucy Aphramor

**Affiliations:** 1 University of California, Davis, and City College of San Francisco, Box S-80, City College of San Francisco, 50 Phelan Avenue, San Francisco, CA 94112, USA; 2 Coventry University, Applied Research Centre in Health and Lifestyle Interventions, Priory Street, Coventry, CV1 1FB, UK; 3 University Hospitals Coventry and Warwickshire NHS Trust, Cardiac Rehab, Cardiology Suite, 1st Floor, East Wing, Walsgrave Hospital, Clifford Bridge Road, Coventry CV2 2DX, UK

## Abstract

Current guidelines recommend that "overweight" and "obese" individuals lose weight through engaging in lifestyle modification involving diet, exercise and other behavior change. This approach reliably induces short term weight loss, but the majority of individuals are unable to maintain weight loss over the long term and do not achieve the putative benefits of improved morbidity and mortality. Concern has arisen that this weight focus is not only ineffective at producing thinner, healthier bodies, but may also have unintended consequences, contributing to food and body preoccupation, repeated cycles of weight loss and regain, distraction from other personal health goals and wider health determinants, reduced self-esteem, eating disorders, other health decrement, and weight stigmatization and discrimination. This concern has drawn increased attention to the ethical implications of recommending treatment that may be ineffective or damaging. A growing trans-disciplinary movement called Health at Every Size (HAES) challenges the value of promoting weight loss and dieting behavior and argues for a shift in focus to weight-neutral outcomes. Randomized controlled clinical trials indicate that a HAES approach is associated with statistically and clinically relevant improvements in physiological measures (e.g., blood pressure, blood lipids), health behaviors (e.g., eating and activity habits, dietary quality), and psychosocial outcomes (such as self-esteem and body image), and that HAES achieves these health outcomes more successfully than weight loss treatment and without the contraindications associated with a weight focus. This paper evaluates the evidence and rationale that justifies shifting the health care paradigm from a conventional weight focus to HAES.

## Introduction

Concern regarding "overweight" and "obesity" is reflected in a diverse range of policy measures aimed at helping individuals reduce their body mass index (BMI)^1^. Despite attention from the public health establishment, a private weight loss industry estimated at $58.6 billion annually in the United States [1], unprecedented levels of body dissatisfaction [2] and repeated attempts to lose weight [3,4], the majority of individuals are unable to maintain weight loss over the long term and do not achieve the putative benefits of improved morbidity and mortality [5]. Concern has arisen that this weight focused paradigm is not only ineffective at producing thinner, healthier bodies, but also damaging, contributing to food and body preoccupation, repeated cycles of weight loss and regain, distraction from other personal health goals and wider health determinants, reduced self-esteem, eating disorders, other health decrement, and weight stigmatization and discrimination [6-8]. As evidence-based competencies are more firmly embedded in health practitioner standards, attention has been given to the ethical implications of recommending treatment that may be ineffective or damaging [5,9].

A growing trans-disciplinary movement called Health at Every Size^SM ^(HAES)^2 ^shifts the focus from weight management to health promotion. The primary intent of HAES is to support improved health behaviors for people of all sizes without using weight as a mediator; weight loss may or may not be a side effect.

HAES is emerging as standard practice in the eating disorders field: The Academy for Eating Disorders, Binge Eating Disorder Association, Eating Disorder Coalition, International Association for Eating Disorder Professionals, and National Eating Disorder Association explicitly support this approach [10]. Civil rights groups including the National Association to Advance Fat Acceptance and the Council on Size and Weight Discrimination also encourage HAES. An international professional organization, the Association for Size Diversity and Health, has developed, composed of individual members across a wide span of professions who are committed to HAES principles.

### Health at Every Size: A Review of Randomized Controlled Trials

Several clinical trials comparing HAES to conventional obesity treatment have been conducted. Some investigations were conducted before the name "Health at Every Size" came into common usage; these earlier studies typically used the terms "non-diet" or "intuitive eating" and included an explicit focus on size acceptance (as opposed to weight loss or weight maintenance). A Pub Med search for "Health at Every Size" or "intuitive eating" or "non-diet" or "nondiet" revealed 57 publications. Randomized controlled trials (RCTs) were vetted from these publications, and additional RCTs were vetted from their references. Only studies with an explicit focus on size acceptance were included.

Evidence from these six RCTs indicates that a HAES approach is associated with statistically and clinically relevant improvements in physiological measures (e.g. blood pressure, blood lipids), health behaviors (e.g. physical activity, eating disorder pathology) and psychosocial outcomes (e.g, mood, self-esteem, body image) [11-20]. (See Table 1.) All studies indicate significant improvements in psychological and behavioral outcomes; improvements in self-esteem and eating behaviors were particularly noteworthy [11-14,16,17,19,20]. Four studies additionally measured metabolic risk factors and three of these studies indicated significant improvement in at least some of these parameters, including blood pressure and blood lipids [11,12,16,17,19,20]. No studies found adverse changes in any variables.

**Table 1:** Randomized controlled HAES studies reported in peer-reviewed journals

Investigation	**Group type**^**a **^**(n)**	Population	Number of treatment sessions	Follow-up (number of weeks post treatment)	Attrition	Improvements			Decre-ments
							
						Physio-logic	Health behaviors	Psycho-social	
**Provencher, et al., 2009**[17]** and 2007**[20]	**HAES **(n = 48); social support (n = 48); control (n = 48)	Overweight and obese women	15	26	8%; 19%; 21%	Not evaluated	Eating behaviors	Not evaluated	None

**Bacon et al, 2005 **[11]** and 2002**[19]	**HAES **(n = 39); diet (n = 39)	Obese women, chronic dieters	30	52	8%; 42%	LDL, systolic blood pressure	Activity, binge eating	Self esteem, depression, body dissatisfact-ion, body image, interoceptive awareness	None

**Rapaport et al., 2000**[16]	**Modified cognitive-behavioral treatment **(n= 37); cognitive behavioral treatment (n= 38)	Overweight and obese women	10	52	16%; 16%	Total cholesterol^**b**^, LDL cholesterol^**b**^, systolic blood pressure^**b**^, diastolic blood pressure^**b**^	Activity^**b**^, dietary quality^**b**^	Emotional well-being^**b**^, distress^**b**^	None

**Ciliska, 1998**[12]	**Psycho-educational **(n = 29); education only (n = 26), waitlist control (n = 23)	Obese women	12	52	14%; 23%; 41%	Diastolic blood pressure	Binge eating	Self-esteem, body dissatisfact-ion, depression	None

**Goodrick et al., 1998**[13]	**Nondiet **(n = 62); diet (n = 65); wait-list control (n = 58)	Overweight and obese women, binge-eaters	50	78	Not reported	Not evaluated	Binge-eating, exercise^**b**^	Not evaluated	None

**Tanco, et al., 1998**[14]	**Cognitive group treatment **(n = 20); weight loss (n = 21); waitlist control (n = 19)	Obese women	8	26	10%; 10%; 32%	Not evaluated	Not evaluated	Depression, anxiety, eating-related psycho-pathology, perception of self-control	None

^a ^HAES group listed first and in bold. (The names reflect those used in the publication.)

^**b **^Improvement in HAES group, but not statistically different from the control.

A seventh RCT reported at a conference also found significantly positive results [18], as did a non-randomized controlled study [21] and five studies conducted without a control [22-26].

All of the controlled studies showed retention rates substantially higher than, or, in one instance, as high, as the control group, and all of the uncontrolled studies also showed high retention rates. Given the well-documented recidivism typical of weight loss programs [5,27,28] and the potential harm that may arise[29,30], this aspect is particularly noteworthy.

## Assumptions underlying the conventional (weight-focused) paradigm

Dieting and other weight loss behaviors are popular in the general population and widely encouraged in public health policy and health care practice as a solution for the "problem" of obesity. There is increasing concern about the endemic misrepresentation of evidence in these weight management policies [5,8]. Researchers have demonstrated ways in which bias and convention interfere with robust scientific reasoning such that obesity research seems to "enjoy special immunity from accepted standards in clinical practice and publishing ethics" [5,8,31]. This section discusses the assumptions that underlie the current weight-focused paradigm, presenting evidence that contests their scientific merit and challenges the value of promoting weight management as a public health measure.

### Assumption: Adiposity poses significant mortality risk

Evidence: Except at statistical extremes, body mass index (BMI) - or amount of body fat - only weakly predicts longevity [32]. Most epidemiological studies find that people who are overweight or moderately obese live at least as long as normal weight people, and often longer [32-35]. Analysis of the National Health and Nutrition Examination Surveys I, II, and III, which followed the largest nationally representative cohort of United States adults, determined that greatest longevity was in the overweight category [32]. As per the report, published in the Journal of the American Medical Association and reviewed and approved by the Centers for Disease Control and Prevention and the National Cancer Institute, "[this] finding is consistent with other results reported in the literature." Indeed, the most comprehensive review of the research pooled data for over 350,000 subjects from 26 studies and found overweight to be associated with greater longevity than normal weight [36]. More recently, Janssen analyzed data in the elderly (among whom more than 70 percent of all deaths occur) - also from 26 published studies - and similarly found no evidence of excess mortality associated with overweight [37]. The Americans' Changing Lives study came to a similar conclusion, indicating that "when socioeconomic and other risk factors are controlled for, obesity is not a significant risk factor for mortality; and... for those 55 or older, both overweight and obesity confer a significant decreased risk of mortality." [38] The most recent analysis, published in the New England Journal of Medicine, concluded that overweight was associated with increased risk, but only arrived at this conclusion after restricting the analysis by excluding 78 percent of the deaths [39]. They also used a reference category much narrower than the entire "normal weight" category used by most other studies, which also contributed to making the relative risk for overweight higher.

There is a robust pattern in the epidemiological literature that has been named the "obesity paradox" [40,41]: obesity is associated with longer survival in many diseases. For example, obese persons with type 2 diabetes [42], hypertension [43,44], cardiovascular disease[41,45], and chronic kidney disease [46] all have greater longevity than thinner people with these conditions [47-49]. Also, obese people who have had heart attacks, coronary bypass[50], angioplasty[51] or hemodialysis [52] live longer than thinner people with these histories [49]. In addition, obese senior citizens live longer than thinner senior citizens [53].

The idea that "this is the first generation of children that may have a shorter life expectancy than their parents" is commonly expressed in scientific journals [54] and popular press articles [55], even appearing in Congressional testimony by former Surgeon General Richard Carmona [56] and a 2010 report from the White House Task Force on Childhood Obesity[57]. When citation is provided, it refers to an opinion paper published in the New England Journal of Medicine [54], which offered no statistical evidence to support the claim. Life expectancy increased dramatically during the same time period in which weight rose (from 70.8 years in 1970 to 77.8 years in 2005) [58]. Both the World Health Organization and the Social Security Administration project life expectancy will continue to rise in coming decades [59,60].

### Assumption: Adiposity poses significant morbidity risk

Evidence: While it is well established that obesity is *associated *with increased risk for many diseases, causation is less well-established. Epidemiological studies rarely acknowledge factors like fitness, activity, nutrient intake, weight cycling or socioeconomic status when considering connections between weight and disease. Yet all play a role in determining health risk. When studies *do *control for these factors, increased risk of disease disappears or is significantly reduced [61]. (This is less true at statistical extremes.) It is likely that these other factors increase disease risk at the same time they increase the risk of weight gain.

Consider weight cycling as an example. Attempts to lose weight typically result in weight cycling, and such attempts are more common among obese individuals [62]. Weight cycling results in increased inflammation, which in turn is known to increase risk for many obesity-associated diseases [63]. Other potential mechanisms by which weight cycling contributes to morbidity include hypertension, insulin resistance and dyslipidemia [64]. Research also indicates that weight fluctuation is associated with poorer cardiovascular outcomes and increased mortality risk [64-68]. Weight cycling can account for all of the excess mortality associated with obesity in both the Framingham Heart Study [69] and the National Health and Nutrition Examination Survey (NHANES) [70]. It may be, therefore, that the association between weight and health risk can be better attributed to weight cycling than adiposity itself [63].

As another example, consider type 2 diabetes, the disease most highly associated with weight and fat distribution. There is increasing evidence that poverty and marginalization are more strongly associated with type 2 diabetes than conventionally-accepted risk factors such as weight, diet or activity habits [30,71-73]. A large Canadian report produced in 2010, for example, found that low income was strongly associated with diabetes even when BMI (and physical activity) was accounted for [73]. Also, much evidence suggests that insulin resistance is a product of an underlying metabolic disturbance that predisposes the individual to increased fat storage due to compensatory insulin secretion [61,74-78]. In other words, obesity may be an early symptom of diabetes as opposed to its primary underlying cause.

Hypertension provides another example of a condition highly associated with weight; research suggests that it is two to three times more common among obese people than lean people [79]. To what extent hypertension is caused by adiposity, however, is unclear. That BMI correlates more strongly with blood pressure than percent body fat [80] indicates that the association between BMI and blood pressure results from higher lean mass as opposed to fat mass. Also, the association may have more to do with the weight cycling that results from trying to control weight than the actual weight itself [48,81,82]. One study conducted with obese individuals determined that weight cycling was strongly positively associated with incident hypertension [82]. Another study showed that obese women who had dieted had high blood pressure, while those who had never been on a diet had normal blood pressure [67]. Rat studies also show that obese rats that have weight cycled have very high blood pressures compared to obese rats that have not weight cycled [83,84]. This finding could also explain the weak association between obesity and hypertension in cultures where dieting is uncommon[48,85]. Additionally, it is well documented that obese people with hypertension live significantly longer than thinner people with hypertension [43,86-88] and have a lower risk of heart attack, stroke, or early death [45]. Rather than identifying health risk, as it does in thinner people, hypertension in heavier people may simply be a requirement for pumping blood through their larger bodies [89].

It is also notable that the prevalence of hypertension dropped by half between 1960 and 2000, a time when average weight sharply increased, declining much more steeply among those deemed overweight and obese than among thinner individuals [90]. Incidence of cardiovascular disease also plummeted during this time period and many common diseases now emerge at older ages and are less severe [90]. (The notable exception is diabetes, which showed a small, non-significant increase during this time period [90].) While the decreased morbidity can at least in part be attributed to improvements in medical care, the point remains that we are simply not seeing the catastrophic disease consequences predicted to result from the "obesity epidemic."

### Assumption: Weight loss will prolong life

Evidence: Most prospective observational studies suggest that weight loss *increases *the risk of premature death among obese individuals, even when the weight loss is intentional and the studies are well controlled with regard to known confounding factors, including hazardous behavior and underlying diseases [91-96]. Recent review of NHANES, for example, a nationally representative sample of ethnically diverse people over the age of fifty, shows that mortality *increased *among those who lost weight [97].

While many short-term weight loss intervention studies do indicate improvements in health measures, because the weight loss is always accompanied by a change in behavior, it is not known whether or to what extent the improvements can be attributed to the weight loss itself. Liposuction studies that control for behavior change provide additional information about the effects of weight (fat) loss itself. One study which explicitly monitored that there were no changes in diet and activity for 10-12 weeks post abdominal liposuction is a case in point. Participants lost an average of 10.5 kgs but saw no improvements in obesity-associated metabolic abnormalities, including blood pressure, triglycerides, cholesterol, or insulin sensitivity [98]. (Note that liposuction removes subcutaneous fat, not the visceral fat that is more highly associated with disease, and these results should be interpreted carefully.)

In most studies on type 2 diabetes, the improvement in glycemic control is seen within days, before significant weight or fat is lost. Evidence also challenges the assumption that weight loss is associated with improvement in long-term glycemic control, as reflected in HbA1c values [99,100]. One review of controlled weight-loss studies for people with type 2 diabetes showed that initial improvements were followed by a deterioration back to starting values six to eighteen months after treatment, *even when the weight loss was maintained *[101].

Furthermore, health benefits associated with weight loss rarely show a dose response (in other words, people who lose small amounts of weight generally get as much health benefit from the intervention as those who lose larger amounts).

These data suggest that the behavior change as opposed to the weight loss itself may play a greater role in health improvement.

### Assumption: Anyone who is determined can lose weight and keep it off through appropriate diet and exercise

Evidence: Long-term follow-up studies document that the majority of individuals regain virtually all of the weight that was lost during treatment, regardless of whether they maintain their diet or exercise program [5,27]. Consider the Women's Health Initiative, the largest and longest randomized, controlled dietary intervention clinical trial, designed to test the current recommendations. More than 20,000 women maintained a low-fat diet, reportedly reducing their calorie intake by an average of 360 calories per day [102] and significantly increasing their activity [103]. After almost eight years on this diet, there was almost no change in weight from starting point (a loss of 0.1 kg), and average waist circumference, which is a measure of abdominal fat, had *increased *(0.3 cm) [102].

A panel of experts convened by the National Institutes of Health determined that "one third to two thirds of the weight is regained within one year [after weight loss], and almost all is regained within five years." [28] More recent review finds one-third to two-thirds of dieters regain more weight than was lost on their diets; "In sum," the authors report, "there is little support for the notion that diets lead to lasting weight loss or health benefits [5]." Other reviews demonstrate the unreliability of conventional claims of sustained weight loss [104,105]. There is a paucity of long term data regarding surgical studies, but emerging data indicates gradual post-surgery weight regain as well [106,107]. Weight loss peaks about one year postoperative, after which gradual weight regain is the norm.

### Assumption: The pursuit of weight loss is a practical and positive goal

Evidence: As discussed earlier, weight cycling is the most common result of engaging in conventional dieting practices and is known to increase morbidity and mortality risk. Research identifies many other contraindications to the pursuit of weight loss. For example, dieting is known to reduce bone mass, increasing risk for osteoporosis [108-111]; this is true even in an obese population, though obesity is typically associated with reduced risk for osteoporosis[108]. Research also suggests that dieting is associated with increased chronic psychological stress and cortisol production, two factors known to increase disease risk [112]. Also, there is emerging evidence that persistent organic pollutants (POPs), which bioaccumulate in adipose tissue and are released during its breakdown, can increase risk of various chronic diseases including type 2 diabetes [113,114], cardiovascular disease [115] and rheumatoid arthritis [116]; two studies document that people who have lost weight have higher concentration of POPs in their blood [117,118]. One review of the diabetes literature indicates "that obese persons that (sic) do not have elevated POPs are not at elevated risk of diabetes, suggesting that the POPs rather than the obesity per se is responsible for the association" [114].

Positing the value of weight loss also supports widespread anxiety about weight [119,120]. Evidence from the eating disorder literature indicates an emphasis on weight control can promote eating disordered behaviors [7]. Prospective studies show that body dissatisfaction is associated with binge eating and other eating disordered behaviors, lower levels of physical activity and increased weight gain over time [121,122]. Many studies also show that dieting is a strong predictor of future weight gain [66,123-128].

Another unintended consequence of the weight loss imperative is an increase in stigmatization and discrimination against fat individuals. Discrimination based on weight now equals or exceeds that based on race or gender [129]. Extensive research indicates that stigmatizing fat demotivates, rather than encourages, health behavior change [130]. Adults who face weight stigmatization and discrimination report consuming increased quantities of food [131-134], avoiding exercise [133,135-137], and postponing or avoiding medical care (for fear of experiencing stigmatization) [138]. Stigmatization and bias on the part of health care practitioners is well-documented, resulting in lower quality care [139,140].

### Assumption: The only way for overweight and obese people to improve health is to lose weight

Evidence: That weight loss will improve health over the long-term for obese people is, in fact, an untested hypothesis. One reason the hypothesis is untested is because no methods have proven to reduce weight long-term for a significant number of people. Also, while normal weight people have lower disease incidence than obese individuals, it is unknown if weight loss in individuals already obese reduces disease risk to the same level as that observed in those who were never obese [91,93].

As indicated by research conducted by one of the authors and many other investigators, most health indicators can be improved through changing health behaviors, regardless of whether weight is lost [11]. For example, lifestyle changes can reduce blood pressure, largely or completely independent of changes in body weight [11,141-143]. The same can be said for blood lipids [11,143-145]. Improvements in insulin sensitivity and blood lipids as a result of aerobic exercise training have been documented even in individuals who *gained *body fat during the intervention [145,146].

### Assumption: Obesity-related costs place a large burden on the economy, and this can be corrected by focused attention to obesity treatment and prevention

Evidence: The health cost attributed to obesity in the United States is currently estimated to be $147 billion annually [147] and this cost estimate has been used to justify efforts at obesity treatment and prevention. Although this estimate has been granted credence by health experts, the word "estimate" is important to note: as the authors state, most of the cost changes are not "statistically different from zero." Also, the estimate fails to account for many potentially confounding variables, among them physical activity, nutrient intake, history of weight cycling, degree of discrimination, access to (quality) medical care, etc. All are independently correlated with both weight and health and could play a role in explaining the costs associated with having a BMI over 30. Nor does it account for costs associated with unintended consequences of positing the value of a weight focus, which may include eating disorders, diet attempts, weight cycling, reduced self-esteem, depression, and discrimination.

Because BMI is considered a risk factor for many diseases, obese persons are automatically relegated to greater testing and treatment, which means that positing BMI as a risk factor results in increased costs, regardless of whether BMI itself is problematic. Yet using BMI as a proxy for health may be more costly than addressing health directly. Consider, for example, the findings of a study which examined the "healthy obese" and the "unhealthy normal weight" populations [148]. The study identified six different risk factors for cardiometabolic health and included subjects in the "unhealthy" group if they had two or more risk factors, making it a more stringent threshold of health than that used in categorizing metabolic syndrome or diabetes. The study found a substantial proportion of the overweight and obese population, at every age, who were healthy and a substantial proportion of the "normal weight" group who were unhealthy. Psychologist Deb Burgard examined the costs of overlooking the normal weight people who need treatment and over-treating the obese people who do not (personal communication, March 2010). She found that BMI profiling overlooks 16.3 million "normal weight" individuals who are not healthy and identifies 55.4 million overweight and obese people who are not ill as being in need of treatment (see Table 2). When the total population is considered, this means that 31 percent of the population is mis-identified when BMI is used as a proxy for health.

**Table 2:** Cost of Using BMI as a Proxy for Health^a^

		Abnormal cardiometabolic profile	Normal cardiometabolic profile	TOTAL
Untreated	"Normal" weight (BMI = 18.5 - 24.9)	23.5% (16.3 million people)^b^	76.5% (53.0 million people)	100% (69.3 million people)

Treated	"Overweight" (BMI = 25.0 - 29.9)	48.7% (34.1 million people)	51.3% (35.9 million people)^c^	100% (70.0 million people)
	
	"Obese" (BMI ≥ 30.0)	68.3% (42.0 million people)	31.7% (19.5 million people)^c^	100% (61.5 million people)

**TOTAL**		46% 92.4 million people	54% 108.4 million people	100% 200.8 million people

^a^Based on study by Wildman et al. [148].

^b^**False negative**: 16.3 million of 92.4 million (17.6%) who have abnormal cardiometabolic profile are overlooked

^c^**False positive**: 55.4 million of 131.5 million (42%) are identified as ill who are not

The weight bias inherent in BMI profiling may actually result in higher costs and sicker people. As an example, consider a 2009 study published in the American Journal of Public Health (96). The authors compared people of similar age, gender, education level, and rates of diabetes and hypertension, and examined how often they reported feeling sick over a 30-day period. Results indicated that body image had a much bigger impact on health than body size. In other words, two equally fat women would have very different health outcomes, depending on how they felt about their bodies. Likewise, two women with similar body insecurities would have similar health outcomes, even if one were fat and the other thin. These results suggest that the stigma associated with being fat is a major contributor to obesity-associated disease. BMI and health are only weakly related in cultures where obesity is not stigmatized, such as in the South Pacific [48,149].

## Health at every size: shifting the paradigm from weight to health

This section explains the rationale supporting some of the significant ways in which the HAES paradigm differs from the conventional weight-focused paradigm. The following topics are addressed:

1) HAES encourages body acceptance as opposed to weight loss or weight maintenance;

2) HAES supports reliance on internal regulatory processes, such as hunger and satiety, as opposed to encouraging cognitively-imposed dietary restriction; and

3) HAES supports active embodiment as opposed to encouraging structured exercise.

### Encouraging Body Acceptance

Conventional thought suggests that body discontent helps motivate beneficial lifestyle change [150,151]. However, as discussed previously in the section on the pursuit of weight loss, evidence suggests the opposite: promoting body discontent instead induces harm [122,133,134,152], resulting in less favorable lifestyle choices. A common aphorism expressed in the HAES community is that "if shame were effective motivation, there wouldn't be many fat people." Mounting evidence suggests this belief is unfounded and detrimental[8,152]. Promoting one body size as more favorable than another also has ethical consequences [120], contributing to shaming and discrimination.

Compassion-focused behavior change theory emerging from the eating disorders field suggests that self-acceptance is a cornerstone of self-care, meaning that people with strong self-esteem are more likely to adopt positive health behaviors [153,154]. The theory is borne out in practice: HAES research shows that by learning to value their bodies as they are right now, even when this differs from a desired weight or shape or generates ambivalent feelings, people strengthen their ability to take care of themselves and sustain improvements in health behaviors [8,11].

Critics of HAES express concern that encouraging body acceptance will lead individuals to eat with abandon and disregard dietary considerations, resulting in weight gain. This has been disproven by the evidence; no randomized controlled HAES study has resulted in weight gain, and all studies that report on dietary quality or eating behavior indicate improvement or at least maintenance [11,14-23]. This is in direct contrast to dieting behavior, which is associated with weight gain over time [66,123-128].

### Supporting Intuitive Eating

Conventional recommendations view conscious efforts to monitor and restrict food choices as a necessary aspect of eating for health or weight control [155]. The underlying belief is that cognitive monitoring is essential for keeping appetite under control and that without these injunctions people would make nutritionally inadvisable choices, including eating to excess. The evidence, however, disputes the value of encouraging external regulation and restraint as a means for weight control: several large scale studies demonstrate that eating restraint is actually associated with weight *gain *over time [66,123-126].

In contrast, HAES teaches people to rely on internal regulation, a process dubbed intuitive eating [156], which encourages them to increase awareness of their body's response to food and learn how to make food choices that reflect this "body knowledge." Food is valued for nutritional, psychological, sensual, cultural and other reasons. HAES teaches people to make connections between what they eat and how they feel in the short- and medium-term, paying attention to food and mood, concentration, energy levels, fullness, ease of bowel movements, comfort eating, appetite, satiety, hunger and pleasure as guiding principles.

The journey towards adopting intuitive eating is typically a process one engages in over time. Particularly for people with a long history of dieting, other self-imposed dietary restriction, or body image concerns, it can feel very precarious to let go of old habits and attitudes and risk trying new ways of relating to food and self. Coming to eat intuitively happens gradually as old beliefs about food, nutrition and eating are challenged, unlearned and replaced with new ones.

A large popular literature has accumulated that supports individuals in developing intuitive eating skills [8,156-160]. (Intuitive eating is also known in the literature as "attuned eating" or "mindful eating." Note that intuitive eating is sometimes promoted as a means to weight loss and in that context is inconsistent with a HAES approach.)

There is considerable evidence that intuitive eating skills can be learned [11,18,161], and that intuitive eating is associated with improved nutrient intake [162], reduced eating disorder symptomatology [17,18,163-165] - and not with weight gain [11,13,16-18]. Several studies have found intuitive eating to be associated with lower body mass [162,163,166,167].

### Supporting Active Embodiment

HAES encourages people to build activity into their day-to-day routines and focuses on helping people find enjoyable ways of being active. The goal is to promote well-being and self-care rather than advising individuals to meet set guidelines for frequency and intensity of exercise. Active living is promoted for a range of physical, psychological and other synergistic benefits which are independent of weight loss. Myths around weight control and exercise are explicitly challenged. Physical activity is also used in HAES as a way of healing a sense of body distrust and alienation from physicality that may be experienced when people are taught to over-ride embodied internal signals in pursuit of externally derived goals, such as commonly occurs in dieting. In addition, some HAES programs have used physical activity sessions, along with other activities such as art and relaxation, to further a community development agenda, creating volunteer, training and employment opportunities and addressing issues of isolation, poor self-esteem and depression among course participants.

## Clinical Ethics

There are serious ethical concerns regarding the continued use of a weight-centered paradigm in current practice in relation to beneficence and nonmaleficence. Beneficence concerns the requirement to effect treatment benefit. There is a paucity of literature to substantiate that the pursuit of weight control is beneficial, and a similar lack of evidence to support that weight loss is maintained over the long term or that programs aimed at prevention of weight gain are successful. Nonmaleficence refers to the requirement to do no harm. Much research suggests damage results from a weight-centered focus, such as weight cycling and stigmatization. Consideration of several dimensions of ethical practice - veracity, fidelity, justice and a compassionate response - suggests that the HAES paradigm shift may be required for professional ethical accountability [168].

## Public Health Ethics

The new public health ethics advocates scrutiny of the values and structure of medical care, recognizing that the remedy to poor health and health inequalities does not lie solely in individual choices.

This ethicality has been adopted by HAES in several ways. HAES academics have highlighted the inherent limitations of an individualistic approach to conceptualizing health. Individual self-care is taken as a starting point for HAES programs, but, unlike more conventional interventions, the HAES ethos recognizes the structural basis of health inequities and understands empowerment as a process that effects collective change in advancing social justice [169]. HAES advocates have also stressed the need for action to challenge the thinness privilege and to better enable fat people's voices to be heard in and beyond health care [8,170].

The hallmark theme of the new public health agenda is that it emphasizes the complexity of health determinants and the need to address systemic health inequities in order to improve population-wide health outcomes and reduce health disparities, making use of the evidence on the strong relationship between a person's social positioning and their health. For example, research since the 1950s has documented huge differences in cardiac health between and across socioeconomic gradients which has come to be recognized as arising from disparities in social standing and is articulated as the status syndrome [171]. Since weight tracks closely with socioeconomic class, obesity is a particularly potent marker of social disparity [172].

There is extensive research documenting the role of chronic stress in conditions conventionally described as obesity-associated, such as hypertension, diabetes and coronary heart disease [173]. These conditions are mediated through increased metabolic risk seen as raised cholesterol, raised blood pressure, raised triglycerides and insulin resistance. The increase in metabolic risk can in part be explained by a change in eating, exercise and drinking patterns attendant on coping with stress. However, changes in health behaviors do not fully account for the metabolic disturbances. Instead, stress itself alters metabolism independent of a person's lifestyle habits [174]. Thus, it has been suggested that psychological distress is the antecedent of high metabolic risk [175], which indicates the need to ensure health promotion policies utilize strategies known to reduce, rather than increase, psychological stress. In addition to the impact of chronic stress on health, an increasing body of international research, discussed earlier, recognizes particular pathways through which weight stigmatization and discrimination impact on health, health-seeking behaviors, and quality of health care [125-133].

Policies which promote weight loss as feasible and beneficial not only perpetuate misinformation and damaging stereotypes [176], but also contribute to a healthist, moralizing discourse which mitigates against socially-integrated approaches to health [155,168,177,178]. While access to size acceptance practitioners can ameliorate the harmful effects of discrimination in health care for individuals, systemic change is required to address the iatrogenic consequences of institutional size discrimination in and beyond health care, discrimination that impacts on people's opportunities and health.

Quite aside from the ethical arguments underscoring inclusive, non-discriminatory health care and civil rights, there are plausible metabolic pathways through which reducing weight stigma, by reducing inequitable social processes, can help alleviate the burden of poor health.

## Conclusion

From the perspective of efficacy as well as ethics, body weight is a poor target for public health intervention. There is sufficient evidence to recommend a paradigm shift from conventional weight management to Health at Every Size. More research that considers the unintended consequences of a weight focus can help to clarify the associated costs and will better allow practitioners to challenge the current paradigm. Continued research that includes larger sample sizes and more diverse populations and examines how best to deliver a Health at Every Size intervention, customized to specific populations, is called for.

We propose the following guidelines, which are supported by the Association for Size Diversity and Health (ASDAH), to assist professionals in implementing HAES. Our proposed guidelines are modified, with permission, from guidelines developed by the Academy for Eating Disorders for working with children [7].

• Interventions should meet ethical standards. They should focus on health, not weight, and should be referred to as "health promotion" and not marketed as "obesity prevention." Interventions should be careful to avoid weight-biased stigma, such as using language like "overweight" and "obesity."

• Interventions should seek to change major determinants of health that reside in inequitable social, economic and environmental factors, including all forms of stigma and oppression.

• Interventions should be constructed from a holistic perspective, where consideration is given to physical, emotional, social, occupational, intellectual, spiritual, and ecological aspects of health.

• Interventions should promote self-esteem, body satisfaction, and respect for body size diversity.

• Interventions should accurately convey the limited impact that lifestyle behaviors have on overall health outcomes.

• Lifestyle-oriented elements of interventions that focus on physical activity and eating should be delivered from a compassion-centered approach that encourages self-care rather than as prescriptive injunctions to meet expert guidelines.

• Interventions should focus only on modifiable behaviors where there is evidence that such modification will improve health. Weight is not a behavior and therefore not an appropriate target for behavior modification.

• Lay experience should inform practice, and the political dimensions of health research and policy should be articulated.

These guidelines outline ways in which health practitioners can shift their practice towards a HAES approach and, in so doing, uphold the tenets of their profession in providing inclusive, effective, and ethical care consistent with the evidence base.

## Appendix

^1^Critics challenge the value of using BMI terminology, suggesting that BMI is a poor determinant of health and the categories medicalize and pathologize having a certain body. We accept this argument; we have used "overweight" and "obese" throughout this paper when necessary to report research where these categories were used. We recognize, however, that "normal" does not reflect a normative or optimal value; that "overweight" falsely implies a weight over which one is unhealthy; and that the etymology of the word "obese" mistakenly implies that a large appetite is the cause.

^2 ^Health at Every Size/HAES is a pending trademark of the Association for Size Diversity and Health.

## Conflict of interests Disclosure

Linda Bacon and Lucy Aphramor are HAES practitioners. Both also speak and write on the topic of Health at Every Size and sometimes receive financial remuneration for this work.

## Authors' contributions

LB initiated the collaboration. Both authors contributed to conceptualizing and drafting the review. LB was lead researcher and undertook the systematic review and designed and completed the tables. Both authors approved the final manuscript.
