# Weight Science: Evaluating the evidence for a paradigm shift

**DOI:** 10.1186/1475-2891-10-69

**Published:** 2011-06-17

**Authors:** Linda Bacon, Lucy Aphramor

**Affiliations:** 1 University of California, Davis, and City College of San Francisco, Box S-80, City College of San Francisco, 50 Phelan Avenue, San Francisco, CA 94112, USA; 2 Coventry University, Applied Research Centre in Health and Lifestyle Interventions, Priory Street, Coventry, CV1 1FB, UK; 3 University Hospitals Coventry and Warwickshire NHS Trust, Cardiac Rehab, Cardiology Suite, 1st Floor, East Wing, Walsgrave Hospital, Clifford Bridge Road, Coventry CV2 2DX, UK

## Correction

### Erratum

Since publication of this article [1], it has come to our attention that there is an error in the section discussing assumptions about obesity-related costs. Table 2 is correct, indicating that 36% of the population is misidentified when BMI is considered, but there is a typographical error in the text which reported it as 31%.
